# Harnessing artificial intelligence for skin-related neglected tropical diseases (skin NTDs): opportunities, challenges and future directions

**DOI:** 10.1093/inthealth/ihaf068

**Published:** 2025-07-01

**Authors:** Rie R Yotsu, Sebastine Oseghae Oiwoh, Hawah Mbali, Niraj Parajuli, Wendemagegn Enbiale, José A Ruiz-Postigo

**Affiliations:** Department of Tropical Medicine and Infectious Disease, Tulane University, 1440 Canal Street, New Orleans, LA 70112, USA; Department of Dermatology, National Center for Global Health and Medicine, Japan Institute for Health Security, 1-21-1 Toyama, Shinjuku-ku, Tokyo 162-8655, Japan; Dermatology and Venereology Unit, Department of Internal Medicine, Irrua Specialist Teaching Hospital, KM 87 Benin Auchi Road, Irrua, Edo State 310115, Nigeria; Department of Internal Medicine, Ambrose Alli University, Ekpoma, Edo State 310104, Nigeria; Mzuzu Central Hospital, Ministry of Health, Malawi Government, P/Bag 2 Luwinga, Mzuzu, Malawi; Regional Dermatology Training Center, Department of Dermatology, Kilimanjaro Christian Medical Center and Department of Dermatology, Kilimanjaro Christian Medical University College, P.O. Box 8332 Moshi, Kilimanjaro, Tanzania; National Academy of Medical Sciences, Bir Hospital, Kathmandu 44600, Nepal; Department of Dermatovenereology, College of Medicine and Health Sciences, Bahir Dar University, Sabatamit Campus, Bahir Dar, Ethiopia; Collaborative Research and Training Center for Neglected Tropical Diseases, Arba Minch University, Nechsar Campus, Arba Minch, Ethiopia; Global Neglected Tropical Diseases Programme, World Health Organization, 20 Avenue Appia, CH-1211, Geneva 27, Switzerland

Skin-related neglected tropical diseases (skin NTDs) are a subset of NTDs recognized by the World Health Organization (WHO) that primarily present with skin manifestations. In 2022, the WHO introduced an integrated approach for skin NTDs to promote resource sharing and coordinated efforts for more effective disease control.^1^ This group of diseases includes Buruli ulcer, cutaneous leishmaniasis, leprosy, mycetoma, noma, scabies and yaws, among others, many of which can cause chronic wounds, swellings and severe skin deformities with possible amputations. If left undiagnosed and untreated, they can lead to life-long disfigurement and disability, significantly impacting the quality of life of affected individuals through economic loss and social stigma. This is notwithstanding the fatality that may complicate many of them. These realities call for a more intentional and coordinated approach to strengthen both patient care and public health strategies for prevention, control and elimination.

Skin NTDs disproportionately affect communities in low-resource settings, where access to dermatologists and specialized care is extremely limited. For example, in many African countries there is less than one dermatologist per million people, with most concentrated in urban centres, leaving rural communities, where the burden is highest, without access to specialized care.^2^ Compounding this challenge is the lack of field-friendly point-of-care diagnostic tests. Currently the only skin NTD with an established point-of-care test is yaws, which utilizes a diagnostic developed for syphilis (Dual Path Platform testing, Chembio Diagnostics, Medford, NY, USA) due to the shared treponemal aetiology. These diagnostic gaps limit timely case detection and treatment initiation, further exacerbating the burden of these diseases.

Artificial intelligence (AI) is revolutionizing healthcare and is a promising technology to strengthen skin NTD detection and management. Broadly, AI encompasses a range of technologies, including machine learning (ML), deep learning (DL), large language models (LLMs) and generative AI, each with different applications depending on the context. As smartphone use and internet access continues to grow, even in low-resource settings, smartphones equipped with AI tools have the potential to provide real-time diagnostic support at the point of care.^3^ Unlike traditional diagnostic tools that are typically disease-specific, AI has the ability to assist in the diagnosis of a range of diseases simultaneously when properly used.^4^ This aligns not only with the current approach for skin NTD integration, but also with the needs of affected communities where the overall burden of skin diseases is high with limited diagnostic and treatment access.

Despite these promising opportunities, progress in AI applications for skin NTDs has been relatively slow. At the First WHO Global Meeting on Skin NTDs in Geneva in March 2023, dermatologists and AI specialists highlighted several persistent barriers, including the limited number and the lack of high-quality skin image datasets to train ML, particularly from populations in low- and middle-income countries (LMICs) where the burden of skin NTDs is highest. The clinical presentations of cutaneous conditions vary across different skin tones^5,6^ and the clinical presentation of some of the skin NTDs varies across geographical regions. This variation can lead to underrepresentation and performance gaps in AI models. The WHO guidance on AI ethics and governance emphasizes the risks of bias related to AI in areas of ethnicity, race and colour.^7^ Addressing such biases is essential to ensure equitable and context-appropriate AI applications. Moreover, these tools must be evaluated within the realities of LMIC health systems and adapted to the diversity of affected populations.

Several pioneering efforts have explored AI applications for skin NTDs. The WHO's SkinNTDs app incorporates a visual classifier to support the detection and screening of 12 skin NTDs and 24 common skin conditions.^8^ A preliminary study involving 605 patients across five counties in Kenya showed approximately 80% sensitivity, with positive feedback from front-line health workers on usability.^9^ Notably, >90% of images in the app's photo library belong to individuals with dark skin tones (Fitzpatrick IV–VI). Another example is VisualDX, an AI-supported clinical decision support platform for dermatology that includes several skin NTDs in their database.^10^ With access to >47 000 high-quality medical images, including 28.5% depicting individuals with dark skin, VisualDx enables rapid construction of differential diagnoses at the point of care. The platform integrates text-based expert knowledge with visual pattern recognition using images, Sympticons and illustrations. Its mobile tool, DermExpert, leverages ML for real-time diagnostic support. Both platforms also serve as valuable training resources for non-specialist healthcare workers.

While AI presents exciting opportunities for skin NTD control, it is crucial to acknowledge its limitations. Diagnosis is the entry point to care—without an accurate diagnosis, people affected by skin NTDs cannot receive appropriate care. AI can support screening and serve as a clinical decision support tool, but it cannot replace a confirmatory diagnosis. Even experienced dermatologists do not rely solely on visual assessment; patient history, physical examination and laboratory tests (including microscopic and PCR-based methods) are often necessary to establish a diagnosis. A pilot study in Côte d'Ivoire explored the potential of AI-based tools for detecting skin NTDs using prospectively collected data from a teledermatology project.^11^ The study highlighted the importance of high-quality, annotated images and the inclusion of confirmed cases—such as polymerase chain reaction–positive Buruli ulcer—to enhance model accuracy and reliability. Emerging technologies that combine different imaging modalities, such as multispectral or thermal imaging, may offer further improvements in disease detection.^12,13^

AI's contributions in skin NTD control may go beyond screening and diagnosis. AI-powered image analysis may track disease progression, providing objective assessments of disease evolution and treatment response. AI can also aid in triaging severe cases, such as malignancy formation in chronic ulcers. Additional uses include data quality improvement techniques to enhance image standardization, reducing inconsistencies in lighting, resolution and skin tone representation and improving AI model performance across diverse populations. It may also contribute to broader public health efforts, and AI-driven epidemiological modelling can help identify high-risk regions, predict outbreaks and optimize resource allocation for skin NTD control.

However, several critical challenges must be addressed. First, there is limited availability of digital data for skin NTDs in diverse populations for training machine models. Second, AI models must be transparent and interpretable, providing confidence scores and rationales for their predictions to support clinical decision-making. Third, patient privacy is a significant concern, particularly for image-based ML and downstream deployment. Finally, the digital divide poses major barriers in resource-limited settings, where many countries still rely on paper-based or non-user-friendly systems, limiting the ability to collect, manage and utilize data. Overcoming this requires broader infrastructure transformation.

Addressing these challenges requires a multipronged approach. Policymakers and global health organizations must establish regulatory frameworks that ensure responsible AI development, safeguard ethical data use and ensure equitable access, building upon existing guidelines such as by the WHO.^7^ However, current guidelines often remain high-level and lack specific operational guidance for implementation at country and regional levels. Involving national institutes of technological development is key to ensuring context-specific adaptation, fostering country ownership and integrating AI frameworks across sectors. Moreover, as technologies evolve, so too must the tools we design. Current solutions may not fully capture the complexity of skin NTD diagnosis, surveillance and care. These gaps highlight the need for new or adapted tools tailored to local realities—justifying continued investment and innovation in this space.

In conclusion, AI holds immense potential to transform the diagnosis, management and surveillance of skin NTDs. Given that these diseases are often overlooked in global health priorities, it is essential to ensure that they are not left behind from the benefits of emerging technologies. To fully harness the potential of AI, we must address key challenges related to data quality, integration and ethics and equity. Moving forward, AI-driven innovations, guided by a collaborative, context-sensitive approach, can help bridge healthcare gaps and advance the overarching goal of ‘skin health for all’.

## Data Availability

Not applicable.
